# Supplementary material for: Translating trait to state assessment: The case of grandiose narcissism
Source: PLoS One. 2023 May 1;18(5):e0284649. doi: 10.1371/journal.pone.0284649 (PMC10150985; doi:10.1371/journal.pone.0284649)
Supplement: S1 File — (PDF) [file pone.0284649.s001.pdf]

Appendix

Partially saturated models

Mplus syntax and output can be found on the Open Science Framework via this link.

Table A1

Within-person results of the partially saturated model including a fully saturated model at the between-person level

	Factor loadings		
Adjective	1 (General)	2 (Admiration)	3 (Rivalry)
Within-person			
Glorious[a]	.33***	.56***	-
Envied[a]	.59***	.23*	-
Prestigious[a]	.44***	.51***	-
Brilliant[a]	.38***	.61***	-
Powerful[a]	.30**	.61***	-
Superior[a]	.32***	.55***	-
Contemptuous[b]	.64***	-	.21
Irritated[b]	.34**	-	.65***
Gleeful[b]	.70***	-	.05
Envious[b]	.50***	-	.32**
Resentful[b]	.41***	-	.39***
Angry[b]	.34*	-	.92***

Note. χ^2 (41) = 63.88, CFI = .99, TLI = .96, RMSEA = .02; $SRMR_{within}$ = .05, $SRMR_{between}$ = .00, CFI_{within} = .99, $RMSEA_{within}$ = .03

Table A2

Between-person results of the partially saturated model including a fully saturated model at the within-person level

	Factor loadings		
Adjective	1 (General)	2 (Admiration)	3 (Rivalry)
<u>Between-person</u>			
Glorious[a]	.56***	.72***	-
Envied[a]	.80***	.30***	-
Prestigious[a]	.52***	.80***	-
Brilliant[a]	.50***	.85***	-
Powerful[a]	.51***	.65***	-
Superior[a]	.65***	.70***	-
Contemptuous[b]	.95***	-	-.07
Irritated[b]	.79***	-	.54***
Gleeful[b]	.88***	-	-.13
Envious[b]	.76***	-	.15
Resentful[b]	1.00***	-	-.01
Angry[b]	.75***	-	.67***

Note. χ^2 (42) = 41.68, CFI = 1.00, TLI = 1.00, RMSEA = .00; $SRMR_{within}$ = .00, $SRMR_{between}$ = .03, $CFI_{between}$ = 1.00, $RMSEA_{between}$ = .00
