# Supplementary material for: Translating trait to state assessment: The case of grandiose narcissism
Source: PLoS One. 2023 May 1;18(5):e0284649. doi: 10.1371/journal.pone.0284649 (PMC10150985; doi:10.1371/journal.pone.0284649)
Supplement: Attachment — Submitted filename: Response to Reviewers.docx [file pone.0284649.s003.docx]

Reviewer #1

1. My first comment is a clarification of my first comment on the original submission (which was not clear to the authors). Rather than mentioning “the current/present study” (and it is clear that the authors are referring to this study and not some other study) multiple times throughout the Introduction, just mention it once in the “Current Study” section. The rest of the Introduction should be a rationale for and review of previous research relevant to the current study, not a description of or transition to it.

**Response:** Thank you for clarifying this a bit further. In response to this comment, we slightly revised the structure of the introduction. Specifically, as requested, we used the introduction to review previous relevant research in this area and build and explain the rationale for our study, but not to transition to it. We have now added a new section ‘Current study’ where we integrated all relevant descriptions of the work that we are actually undertaking. We believe that this revised structure indeed helped to improve the overall readability of our introduction.

2. My comment also still stands for the overly long Results section due in part to repeating numbers in tables (e.g., r and omega coefficients, p-values).

**Response**: We have shortened the Results section by removing numbers that were copied from the tables. We also removed some additional clarifications that are not essential to understanding the findings of our study. Note that we would prefer some clarifications (e.g., regarding bifactor indices) given that not everyone will be familiar with the exact meaning of (some of) these statistics.

Reviewer #2

The authors have carefully addressed all of my comments, and I have no further concerns about the paper.

However, I have one minor recommendation before publication concerning the following sentence that the authors added on page 9: “At the state level, Edershile et al. (2019) first tested the structure of grandiose and vulnerable narcissism as a multilevel two-factor model, and consequently modeled grandiose narcissism as a model with one factor at both the within- and between-person level (Edershile & Wright, 2020).”

The word “consequently” implies that the choice of Edershile and Wright (2020) to use a one-factor model at both levels of analysis is a direct consequence of Edershile et al.’s first study (2019), in which they used a two-factor model. However, without providing information on the results (e.g., stating that the two-factor model provided a poor fit, if that was the case), the sentence is not clear.

Since correcting this lack of clarity will be easy, no further reviews are needed from my side. I believe the paper is ready for publication.

**Response:** Thank you for pointing this out. We revised this section to remove this ambiguity from our text.
